# Evaluation of the Performance of Females as Light Infantry Soldiers

**DOI:** 10.1155/2014/572953

**Published:** 2014-08-18

**Authors:** Aharon S. Finestone, Charles Milgrom, Ran Yanovich, Rachel Evans, Naama Constantini, Daniel S. Moran

**Affiliations:** ^1^Department of Orthopaedic Surgery, Assaf Harofeh Medical Center, Zerifin & Sackler School of Medicine, Tel Aviv University, 69978 Tel Aviv, Israel; ^2^Israel Defense Forces Institute of Military Physiology and Heller Institute of Medical Research, Haim Sheba Medical Center, Military P.O. Box 02149, 52621 Ramat Gan, Israel; ^3^Hebrew University Medical School, Ein Kerem, 91120 Jerusalem, Israel; ^4^Bone Health Research Program, U.S. Army Research Institute of Environmental Medicine, Military Performance Division, Natick, MA 01760, USA; ^5^Ariel University, 40700 Ariel, Israel

## Abstract

A few countries permit women to serve in combat roles, but their long term performance in these positions has not been reported. The incidences of overuse injuries and attrition of 85 male and 235 female recruits in a light infantry brigade was followed in a three-year prospective study. Females were shorter (162 cm, CI 161–163 cm) than males (174 cm, CI 173–176), had more body fat (18.9 kg, CI 18.2–19.6 kg) than males (12.6 kg, 11.3–13.8 kg), had lower $\dot{\text{V}}$O_2_max (36.8 mL*·*min^−1^ *·*kg^−1^, CI 35.8–37.78 mL*·*min^−1^ *·*kg^−1^) than males (50.48 mL*·*min^−1^ *·*kg^−1^, CI 48.4 to 52.48 mL*·*min^−1^ *·*kg^−1^), had more stress fractures (21.0%, 95% CI 16.2–26.5%) than males (2.3%, CI 0.3–8.2%), and had more anterior knee pain (41.2%, CI 34.9–47.7%) than males (24.7%, CI 16.0–35.2%). Three-year attrition was 28% CI 22–34% for females and 37% CI 26–48% for males. The females in this study successfully served as light infantry soldiers. Their lower fitness and high incidence of overuse injuries might impede service as regular infantry soldiers.

## 1. Introduction

Opposition to women serving in military combat positions has come both from within the armed forces and from groups in the general society. The arguments are many, including the position that women are not able to adequately perform combat duties. Beginning in the 1970s, Canada, the USA, Taiwan, Israel, New Zealand, and a few European nations gradually began to allow women to serve in more active combat roles. Of these countries, only Israel has compulsory military service for women. The performance of women in these active combat roles has not been well documented and publicized. Military studies have found that female stress fracture risk and overuse injury risk are greater than those of males doing the same training [1, 2]. Other studies have focused on identifying gender specific risk factors for overuse injuries in the military [3–5].

Measurement of the professional performance of combat soldiers is multifactorial. Dropout from combat units can be considerable. It can be secondary to acute injuries, overuse injuries, lack of motivation, sociological reasons, or psychological factors. Several studies have focused on the attrition of females in the armed forces [6–10]. During U.S Army Basic Combat Training, the medical discharge rate for males is 3.3 percent as compared to 8.7 percent for females [11]. At one year, the attrition rate among female U.S. Marine Corp recruits was reported to be 17 percent [6]. The overall attrition rate of female soldiers in the U.S. Marine Corp has been reported to be 1.6 times higher than that of males [7].

In 1995, the Israel Supreme Court ruled that the Israel Ministry of Defense policy of not allowing women entrance into the Israel Air Force pilot's course was solely based on gender and, therefore, constituted unlawful discrimination. This decision initiated radical changes in the combat active service opportunities for women in the Israel Defense Forces (IDF). Today, women serve in combat roles in many IDF units including the light infantry brigade Karakal, antiaircraft units, the Home Front Command, and the Border Police, in the Navy and in the Air Force.

In view of the controversy about females serving in combat positions, we undertook a study to compare the long term performance of females and males in the mixed gender light infantry brigade of the Israeli Defense Forces. We report the results of a prospective study of the incidences of their overuse injuries, attrition, and completion of officers' course over the course of their three years of compulsory service.

## 2. Methods

### 2.1. Subjects

Mandatory military service in Israel is three years for males and two years for females. Before induction, future recruits undergo a battery of tests which rate their psychotechnical skills (maximum score 90) and rank their quality as potential military recruits (maximum score 56). They also are given a health profile which determines whether their health status allows them to serve as combat soldiers or not. Males are assigned to service in Karakal, a mixed gender light infantry brigade from the general induction pool of combat eligible males, but females, in addition to having combat eligible health profiles, need to request and volunteer for the unit, agreeing to extend their service from two to three years. The study subjects are female and male Karakal recruits from three consecutive inductions (September 2004 through July 2005). Karakal basic training is 14 weeks. Completion of this training qualifies recruits as light infantry soldiers.

The study was approved by the IRB's of the Israel Defense Forces Medical Corps, the Sheba Medical Center, and the U.S. Army Research Institute of Environmental Medicine at Natick, MA. All the participants signed informed consent for participation in the study.

### 2.2. Measurements

All subjects in the study had baseline measurements during the first week after their military induction and had subsequent follow-up measurements at the end of basic training 14 weeks later. Anthropometric measurements were made of height (nearest cm), weight (after voiding), external rotation of the hip at 90 degrees of flexion, tibia length (medial knee joint line to the most medially prominent point of the medial malleolus), and body fat percentage (calculated from four skinfold thickness measurements over right biceps, triceps, scapula, and iliac crest, Lange skinfold calipers (Beta Technology, Santa Cruz, CA)) [11–13]. Maximum quadriceps force was measured in kgF, using a quadriceps extension machine. $\dot{\text{V}}$O_2_max (mL*·*min^−1^ *·*kg^−1^) was measured using the modified Bruce treadmill protocol, using peak oxygen uptake (SensorMedics Co., CA, USA) [14–16]. According to the protocol, exercise was increased incrementally until the $\dot{\text{V}}$O_2_ reached a plateau and was not increase further in spite of increased effort.

Recruits' pre- and posttraining physical fitness was assessed in the field with the standard IDF fitness test (originally developed by Oded Bar-Or and since modified) administered by the unit officers during the first two weeks of basic training and subsequently during weeks 14-15 after induction. The test includes a 2 km run (minimum time in minutes) and the maximum number of pushups and situps performed consecutively without rest [15].

Questionnaires were administered relating to preinduction injury (including stress fractures (SF), ankle sprains, and back and knee pain), preinduction sports activity [17], smoking history, motivation, cohesion, and stress [18]. This study also used the Bem Sex Role Inventory (BSRI) [19] to calculate the participants' socially defined degrees of masculinity and femininity. Each participant's scores were dichotomized for both masculinity and femininity by their medians, which further allocated them into the four categories of (a) under the median in both scores (nonsex type), (b) over the median in both scores (androgynous type), (c) below the masculine and over the feminine score (feminine type), and (d) over the masculine and under the feminine score (masculine type). The Lazarus and Folkman Ways of Coping questionnaire [20] was used to assess the participants' coping styles (respondents' cognitive and behavioral efforts when confronting stress: scores for distance, problem solving, acceptance, and growth). The Kobasa Questionnaire [21, 22] was used to measure hardiness, subdivided into three subscales measuring commitment, control, and challenge. Burnout was measured using the Shirom-Melamed Burnout Questionnaire [23]. Self-efficacy was assessed using a specially designed nine-item questionnaire. A six-item stress questionnaire included questions regarding perceived stress, having tools to deal with stress, and the recruits' opinion of whether he/she expected to fulfill three years of service.

Female and male combatants were examined in the field by a team of orthopedic surgeons every 2 to 3 weeks during 16 weeks of basic training and were screened for overuse injuries. Suspected stress fractures were diagnosed by radiography and/or bone scan and were treated according to the IDF protocol [24, 25]. All data were recorded directly into a laptop using an Access database (Microsoft Corp., Redmond, WA). Injuries that occurred after completion of basic training were collected manually from the IDF Computerized Patient Record (a centralized medical file) and inserted directly into the Access database. Data on completion of stages and courses in military service and hospitalizations were taken from the central IDF manpower database and subsequently entered into the Access database.

### 2.3. Statistical Analysis

Data analysis was performed on SAS (SAS Institute Inc., Cary North Carolina, USA). Univariate analysis on nominal variables was performed using chi-square (or Fisher's exact test when expected cell count <5) and on continuous variables using Student's *t*-test or ANOVA (analysis of variance). For changes over the training period, paired *t*-tests and repeated measures ANOVA using GLM (general linear models) were used. Hospital admissions were counted if they were for more than 24 hours. Attrition rates were compared at 18 months and at three years. Survival analysis for combatants in their designated service was performed using PROC LIFETEST (a standard SAS routine). Data were censored at 18 months/three years. Attrition date was set at the earlier of two dates, the subject leaving the unit or receiving a medical profile that made leaving obligatory. Variables, possibly related to stress fracture and attrition in female combatants, were tested using Student's *t*-test, and relevant variables were tested in multivariate analysis using stepwise logistic regression. Significant difference was at *P* < 0.05.

## 3. Results

238 females (94% of those eligible) and 85 males (91% of those eligible) volunteered to participate in the study. 227 out of the 238 females (95%) and 78 out of the 85 males (92%) completed basic training. The baseline anthropometric data for those recruits who completed basic training is presented in Table 1. Females were shorter (162 cm, CI 161–163 cm) than males (174 cm, CI 173–176), had more body fat (18.9 kg, CI 18.2–19.6 kg) than males (12.6 kg, 11.3–13.8 kg), had lower quadriceps strength (82 kgF, CI 78 to 86 kgF) than males (124 kgF, 117 to 131 kgF), and had lower $\dot{\text{V}}$O_2_max (36.8 mL*·*min^−1^ *·*kg^−1^, CI 35.8–37.78 mL*·*min^−1^ *·*kg^−1^) than males (50.48 mL*·*min^−1^ *·*kg^−1^, CI 48.4 to 52.48 mL*·*min^−1^ *·*kg^−1^). The results of their physical fitness tests are presented in Table 2.

On induction, 38% of the females and 49% of males reported smoking (*P* = 0.08). Sixteen percent of females and 23% of males reported that they smoked at least half a pack for more than a year (*P* = 0.2). There was no statistically significant difference in the preinduction sports participation between females (48%) and males (52%).

The mean psychotechnical index of females was 58.2 ± 16.8 and that of males was 57.6 ± 19.7(*P* = 0.8). The quality measurement for females was 51.7 ± 2.8 and for males 51.2 ± 3.7(*P* = 0.2).

During the 14 weeks of basic training, both groups gained weight but the gain was not significant for males. Female soldiers gained 1.3 ± 1.7 kg of lean body mass. On repeated measures analysis, group and phase differences were statistically significantly, with no interaction for weight and $\dot{\text{V}}$O_2_max and interaction for fat percent, fat mass, and lean body mass. The Pearson Correlation Coefficient between induction $\dot{\text{V}}$O_2_max and the 2 km running time was 0.479 for females and 0.372 for males.

**Figure 1:**
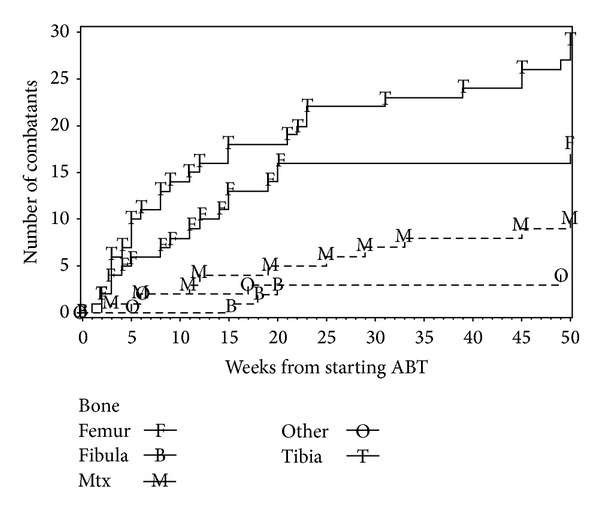
Onset of stress fractures (in weeks from starting basic training) for female soldiers by bone. Fractures after 50 weeks were marked at 50.

The incidence of stress fracture diagnosed during the 36-month period was higher for females (21.0%, 95% CI 16.2–26.5%) than for males (2.3%, CI 0.3–8.2%) (Table 3). The date of onset of pain for those diagnosed with stress fractures is presented in Figure 1. The females had more anterior knee pain (41.2%, CI 34.9–47.7%) than males (24.7%, CI 16.0–35.2%). On univariate analysis, the only variables that were found to have a statistically significant relationship to the incidence of stress fractures among females were the lower BMI (22.0 kg*·*m^−2^ versus 23.3 kg*·*m^−2^, *P* = 0.02) and less body fat (16.9 kg versus 18.7 kg, *P* = 0.04) among those with stress fractures as compared with those without stress fractures. Neither of these factors was significant on multivariate analysis.

At 18 months, 76% of females and 68% of males were still serving as combat soldiers. Three-year attrition was (28%, CI 22–34%) for females and (37% CI 26–48%) for males. The main reasons for not completing service were nonmedical (Table 4). Medical reasons for attrition are detailed in Table 5. Survival analysis demonstrated that female soldiers survived 28.2 ± 0.8 months (mean ± SE) and males 19.4 ± 0.9 months. Females who dropped out had a statistical trend of weaker quadriceps force than their counterparts who remained in the unit (79 kgF versus 87 kgF, *P* = 0.08) and a slower running time (12 : 44 versus 12 : 15, *P* = 0.06) but no difference in $\dot{\text{V}}$O_2_max. Attrition was significantly related to having back pain during training. Females with backache were significantly more likely to drop out (37%) than males (23%), *P* = 0.01. Rates of nongynecological hospital admissions were 12% for females and 16% for males during the 36-month period.

Attrition was associated with a significantly higher response to the question “Do you think the assignment you are undertaking will be stressful?” (4.1 versus 3.3 on a scale of 1 to 5, *P* < 0.0001). Attrition was also associated will significantly lower response to “I am likely to complete 3 years in this assignment” (4.0 versus 4.4 on a scale of 1 to 5, *P* = 0.003). Of the factors derived from the various questionnaires, attrition was significantly associated with an androgen personality trait (respondents with high scores on both masculine and feminine characteristics: androgen 43%, not androgen 22%, and relative risk 2.0 confidence interval 1.2 to 3.1). On multivariate analysis, all above factors entered the model (in decreasing order of statistical significance: expected stress, negative anticipation of assignment completion, androgen trait, and back pain during service).

Five percent of females and one percent of males in the study completed officer's course and became officers. Sixty percent of females and males did reserve army service after completing their three years of mandatory service.

## 4. Discussion

Female soldiers of Karakal had a high rate of attrition, with only seventy two percent remaining as combat soldiers throughout their three years of military service. This retention rate, however, was in fact higher than that of male Karakal soldiers. From this standpoint, the program of female service in the mixed gender light infantry unit Karakal can be considered a success.

From a physical standpoint, the females in Karakal, when compared to the males, began their training at a physiological disadvantage. Their $\dot{\text{V}}$O_2_max measurements and time for a 2 km run were poorer than those of the males. $\dot{\text{V}}$O_2_max in itself is known to be a poor prediction of run performance. The velocity and duration at which subjects can operate at their $\dot{\text{V}}$O_2_max provide better indications of performance. Females were able to do less situps and pushups than males at the beginning of basic training. This deficit remained even after 14 weeks of basic training. The females were also shorter and weighed less than the males. This put them at a disadvantage to the males because they used the same equipment and carried the same base loads while training [26]. The females sustained a 21-percent incidence of stress fractures versus a 2-percent incidence for the males. With the exception of stress fractures and anterior knee pain, which were higher in females, there was no difference in the incidence of the other types of overuse injuries between females and males. In spite of the high incidence of stress fractures and the physical and physiological differences between the women and men of Karakal, their attrition rates because of medical reasons were the same.

The attrition rate for males in Karakal was higher than that of the females principally because of drop out for psychological reasons. The psychological variables specifically measured in this study do not fully explain this large difference. One reason may be because the females in Karakal volunteered specifically for service in Karakal while the males were assigned to the unit from the general pool of males designated for combat service. For females, Karakal is one of the most prestigious army combat units in which they could serve, but, for males, it is one of the least prestigious combat units in which they can serve. The IDF has several systems for evaluating recruits prior to induction. There was no statistically significant difference in the preinduction psychotechnical scores and quality scores between the women and men of Karakal. The psychotechnical scores of the Karakal soldiers were lower than those of regular IDF infantry soldiers. Both the female and male Karakal attrition rates were much higher those of regular IDF infantry soldiers, who have more arduous training. Neither of the psychotechnical nor quality scores were predictors of attrition in this study.

Discharge rates among female American Marine Corp recruits, based on 1999-2000 data, have been reported to be 11.2 percent in basic training, with a one year attrition rate of 17.1 percent [7]. This compares to the 24-percent attrition rate for the females of Karakal at 18 months. Unlike the female Marines, most of the Karakal females who left their unit continued to serve but in noncombat positions. In another American Marine Corp study, based on data from 1997, the female discharge rate was 18.2 percent and the male rate was 11.9 percent during 63 days of Marine Corp basic training at Parris Island [6].

In the current study, 5% percent of the females and one percent of the males completed officer's course and became officers. The IDF is different from the American Army in the fact that officers begin as regular soldiers and therefore data cannot be compared. Those who are identified during their training as being good officer candidates are offered the chance to take an officer's course. Being an officer also requires the soldier to serve for an additional year and not all candidates are willing to do so. About 10% of soldiers in IDF regular infantry units complete officer's course. Since the IDF relies heavily on reserve soldiers, the number of recruits who continue to serve in the reserves after completing mandatory service is important. In this study, 60 percent of both the males and females did reserve army service. This compares with more than 80 percent of regular infantry soldiers.

While this study indicates that women have successfully performed the role of light infantry soldiers in the IDF Karakal unit, the results should not be extrapolated to conclude that they would be similarly successful in a regular infantry unit. While infantry soldiers are expected to be able to carry equipment and supplies sufficient for several days of marching and combat 15–20 kilometers beyond enemy lines, light infantry are not. They are only expected to be able to march 4-5 kilometers with limited combat equipment. They perform most missions and patrols using vehicles. A further difference is the level of fitness. Only 48% of the females and 52% of the males in Karakal reached fitness levels at the end of basic training that would have allowed them to pass minimum IDF infantry soldier standards. The mean $\dot{\text{V}}$O_2_max of the females at the end of basic training (36.8 mL*·*kg^−1^ *·*min^−1^) was lower than the norm for females of their age with average fitness (43 mL*·*kg^−1^ *·*min^−1^) [27].

While some of the Karakal female soldiers could possibly be successful infantry soldiers, there are general inherent physical obstacles. Females have inherently only 70 percent of the lower body strength and 30–50 percent of the upper body strength of comparable sized males [28]. Also, because of hormonal differences, females doing strength training build less muscle bulk than males [29]. Identifying females who could successfully serve in IDF infantry units would require intensive screening and include physical fitness and exertion tests, similar to those required before admission to more elite IDF infantry units. This is especially necessary because while the female recruits in Karakal were volunteers for their unit, most of them did little prior physical activity to prepare themselves for the physical challenges of infantry service [27].

This paper has several limitations. The study compares the performance of females to that of males serving as light infantry soldiers. The study is observational and, therefore, the cohorts could not be matched in any way. The females in Karakal are volunteers to the unit while the males are assigned. The unit has high prestige for the females and low prestige for the males. This results in group bias but does not invalidate the group comparison which is between females and a male cohort who ordinarily serve in such a unit. It does not invalidate the study conclusion that the females perform at least as good as the males in the Karakal unit. Another study weakness is that because the data is pooled from several induction groups there may be variations in methods between groups. Additionally, not all tests were performed on all subjects. This is because, on any individual assessment day, soldiers may have been on limited duty or away from their unit on other assignments. The study supplemented data gathered by the orthopaedic surgeons in the field during basic training by computerized patient records. The active surveillance by the orthopaedists could have led to higher reported injury rates than those which are usually reported.

We conclude that, in general, female participation in the mixed gender light infantry Karakal unit is a success. Females are able to fulfill successfully the combat role of a light infantry soldier. Female soldiers in the unit have lower attrition rates than the males. The higher female incidence of overuse injuries and their lower physical fitness than the males throughout all stages of the Karakal training might represent an impediment to them performing the more arduous training and duties of regular infantry soldiers.

## Figures and Tables

**Table 1:** Anthropometric data (mean ± SD) for female and male Karakal recruits who completed basic training before and after 14 weeks of basic training course.

	Females (*n* = 227)	Males (*n* = 78)	*P* value
Age (year)	19.1 ± 0.6	19.3 ± 1.2	NS
Height (cm)	162 ± 6	175 ± 7	<0.0001
BMI (kg*·*m^−2^)	23.1 ± 3.4	22.8 ± 4.0	NS
Quadriceps force (kgF)	82 ± 24	124 ± 29	<0.0001
Weight (at induction, kg)	60.7 ± 9.9	69.8 ± 13.1	<0.0001
Weight (at week 14, kg)	61.5 ± 9.6	68.9 ± 11.7	<0.0001
ΔWeight (kg)	0.8 ± 2.6∗∗∗	−0.1 ± 5.0^§^	NS
Fat % (at induction)	30.7 ± 4.6	17.3 ± 4.8	<0.0001
Fat % (at week 14)	29.6 ± 4.4	15.9 ± 4.0	<0.0001
ΔFat %	−1.2 ± 2.9∗∗∗	−1.3 ± 2.2∗∗∗	NS
Fat mass (at induction, kg)	18.9 ± 5.5	12.6 ± 5.7	<0.0001
Fat mass (at week 14, kg)	18.5 ± 5.2	11.4 ± 4.4	<0.0001
ΔFat mass (kg)	0.5 ± 2.3∗∗	−0.1 ± 2.3∗∗	NS
Lean body mass (at induction, Kg)	41.7 ± 5.4	57.2 ± 8.1	<0.0001
Lean body mass (at week 14, kg)	43.0 ± 5.3	57.6 ± 7.9	<0.0001
ΔLean body mass (kg)	1.3 ± 1.7∗∗∗	1.1 ± 3.6∗	NS
$\dot{\text{V}}$o_2_max (at induction, mL*·*kg^−1^ *·*min^−1^)	36.8 ± 6.4	50.4 ± 7.8	<0.0001
$\dot{\text{V}}$o_2_max (at week 14, mL*·*kg^−1^ *·*min^−1^)	39.8 ± 5.9	52.4 ± 7.0	<0.0001
Δ$\dot{\text{V}}$o_2_max (mL*·*kg^−1^ *·*min^−1^)	3.0 ± 7.6∗∗∗	2.0 ± 7.6^§^	NS

*P* values for deltas: *P = 0.05, **P < 0.001, ***P < 0.0001, and ^§^NS.

**Table 2:** Before and after 14-week basic training female and male fitness test (mean ± SD).

	Females (*n* = 197)	Males (*n* = 60)	*P* value
2 km run at induction (sec)	739 ± 115	570 ± 101	<0.0001
2 km run at 14 weeks (sec)	667 ± 76.0	533 ± 80.4	<0.0001
ΔRun time (sec)	−72 ± 78∗∗∗	−37 ± 68∗∗	<0.002
Pushups at induction	40 ± 11	41 ± 18	<0.0001
Pushups at 14 weeks	49 ± 7	67 ± 14	<0.0001
ΔPushups	9 ± 13∗∗∗	26 ± 14∗∗∗	<0.0001
Situps at induction	66 ± 27	64 ± 26	NS
Situps at 14 weeks	84 ± 12	86 ± 10	NS
ΔSitups	17 ± 28∗∗∗	22 ± 23∗∗∗	NS

*P* values for deltas: **P < 0.01, ***P < 0.0001.

**Table 3:** Stress fracture (SF) and other overuse injuries of female and male soldiers (%) during 36 months of military service.

	Females (*N* = 238)	Males (*N* = 85)	*P*
Femoral SF	17 (7.1)	1 (1.2)	<0.04
Tibia SF	29 (12.2)	1 (1.2)	<0.003
Fibular SF	3 (1.3)	0	NS
Metatarsal SF	10 (4.2)	0	<0.05
Other SF	4 (1.7)	0	NS
Total SF	50 (21.0)	2 (2.3)	<0.0001
Ant knee pain	98 (41.2)	21 (24.7)	<0.007
Back pain	88 (37.0)	36 (42.3)	NS
Ankle sprain	46 (19.3)	16 (18.8)	NS
Achilles tendinitis	7 (2.9)	3 (3.5)	NS

**Table 4:** Summary of the formal reasons of attrition. Medical and psychological issues needed professional diagnosis and profile change through a formal medical board. Administrative issues refer to all other reasons for attrition including the soldier's request, lack of motivation, lack of physical, social, or mental competency, and family issues preventing engaging in training or service.

Reasons for attrition	Females (*N* = 238)	Males (*N* = 85)	*P* value
Medical issues	18 (7.6%)	6 (7.1%)	NS
Psychological issues	14 (5.9%)	16 (18.8%)	<0.001
Administrative issue	35 (14.7%)	9 (10.6%)	NS

Total attrition	67 (28.2%)	31 (36.5%)	NS
Completed designated service	171 (71.8%)	54 (63.5%)	

**Table 5:** Summary of the formal medical reasons for attrition.

Reasons for attrition	Females (*N* = 238)	Males (*N* = 85)	Total
Back pain	3	1	4
Knee pain	3	1	4
Irritable bowel syndrome	2		2
Pes cavus/Pes planus	2		2
Skin disease	2		2
Asthma	1	1	2
Anaemia	1	1	2
Ankle sprain	1		1
Stress fracture (nondisplaced)	1		1
Fracture (upper extremity)	1		1
Fracture of spine	1		1
Kidney disease		1	1
Ticks (neurological)		1	1

Total	18	6	25

## Dedication

The authors would like to dedicate this paper to the late Ms. Judith Misch, IDF medical informatics; without her assistance, this paper and many others could not have been completed. Her love of soldiers and data fueled her lifelong career in research to improve their health during their service.
